# Cancer therapy-related cardiac dysfunction and the role of cardiovascular imaging: systemic review and opinion paper from the Working Group on Cardio-Oncology of the Korean Society of Cardiology

**DOI:** 10.1186/s44348-024-00014-5

**Published:** 2024-07-30

**Authors:** Iksung Cho, Seng-Chan You, Min-Jae Cha, Hui-Jeong Hwang, Eun Jeong Cho, Hee Jun Kim, Seong-Mi Park, Sung-Eun Kim, Yun-Gyoo Lee, Jong-Chan Youn, Chan Seok Park, Chi Young Shim, Woo-Baek Chung, Il Suk Sohn

**Affiliations:** 1 Division of Cardiology, Severance Cardiovascular Hospital, Yonsei University College of Medicine, Seoul, Republic of Korea; 2 Department of Biomedical Systems Informatics, Yonsei University College of Medicine, Seoul, Republic of Korea; 3 Department of Radiology, Chung-Ang University Hospital, Chung-Ang University College of Medicine, Seoul, Republic of Korea; 4 Department of Cardiology, Kyung Hee University Hospital at Gangdong, Kyung Hee University College of Medicine, Seoul, Republic of Korea; 5 Division of Cardiology, Department of Internal Medicine, Heart and Brain Hospital, Chung-Ang University Gwangmyeong Hospital, Chung-Ang University College of Medicine, Gwangmyeong, Republic of Korea; 6 Division of Medical Oncology, Department of Internal Medicine, Chung-Ang University Hospital, Seoul, Republic of Korea; 7 Division of Cardiology, Department of Internal Medicine, Korea University College of Medicine, Seoul, Republic of Korea; 8 Department of Cardiovascular Medicine, Kangdong Sacred Heart Hospital, Seoul, Republic of Korea; 9 Division of Hematology & Medical Oncology, Department of Internal Medicine, Kangbuk Samsung Hospital, Sungkyunkwan University School of Medicine, Seoul, Republic of Korea; 10 Division of Cardiology, Department of Internal Medicine, College of Medicine, Seoul St. Mary’s Hospital, The Catholic University of Korea, Seoul, Republic of Korea; 11 Division of Cardiology, Department of Internal Medicine, College of Medicine, The Catholic University of Korea, Seoul, Republic of Korea

**Keywords:** Cardio-Oncology, Chemotherapy, Toxicity, Cardiovascular

## Abstract

Cardio-oncology is a critical field due to the escalating significance of cardiovascular toxicity as a side effect of anticancer treatments. Cancer therapy-related cardiac dysfunction (CTRCD) is a prevalent condition associated with cardiovascular toxicity, necessitating effective strategies for prediction, monitoring, management, and tracking. This comprehensive review examines the definition and risk stratification of CTRCD, explores monitoring approaches during anticancer therapy, and highlights specific cardiovascular toxicities linked to various cancer treatments. These include anthracyclines, HER2-targeted agents, vascular endothelial growth factor inhibitors, immune checkpoint inhibitors, chimeric antigen receptor T-cell therapies, and tumor-infiltrating lymphocytes therapies. Incorporating the Korean data, this review offers insights into the regional nuances in managing CTRCD. Using systematic follow-up incorporating cardiovascular imaging and biomarkers, a better understanding and management of CTRCD can be achieved, optimizing the cardiovascular health of both cancer patients and survivors.

## Background

As cancer-related mortality declines steadily due to advances in early cancer detection and anticancer treatment, the importance of anticancer treatment side effects has increased [1–3]. Most importantly, among the various adverse consequences of anticancer therapy, cardiovascular toxicity including arrhythmia, thromboembolic events, atrial fibrillation, and cardiac dysfunction has been recognized [4–6]. In this context, cardio-oncology has been introduced as a subspecialty that involves a team of cancer surgeons, oncologists, hematologists, radiologists, specialist nurses, pharmacists, and cardiologists [7, 8]. The cardio-oncology team offers prevention, monitoring, and treatment for cardiac complications associated with anticancer treatment [9]. Recently, the European Society of Cardiology (ESC) in collaboration with the European Hematology Association (EHA), the European Society for Therapeutic Radiology and Oncology (ESTRO), and the International Cardio-Oncology Society (ICOS) published the first guidelines on cardio-oncology, and the role of the cardio-oncology team has been systematically established [10]. These guidelines have also standardized definitions of chemotherapy-related cardiovascular toxicity, introducing the term "cancer therapy-related cardiovascular toxicity" (CTR-CVT), which includes various specific conditions, such as cancer therapy-related cardiac dysfunction (CTRCD), immune checkpoint inhibitor (ICI) myocarditis, vascular toxicity, arterial hypertension, and cardiac arrhythmia.

Within CTR-CVT, CTRCD is the most prevalent clinical manifestation [11]. The evidence regarding CTRCD continues to increase, especially in light of the evolution of anticancer therapies, such as HER2-positive targeted drugs, proteasome inhibitors, ICIs, and vascular endothelial growth factor (VEGF) inhibitors [12–16]. At the same time, imaging modalities to diagnose and prognosticate CTRCD are evolving including myocardial strain, myocardial work in transthoracic echocardiography (TTE), cardiac computed tomography (CT), and cardiac magnetic resonance[17–21]. The present review of current data and professional guidelines proposes potential strategies for predicting, monitoring, and tracking CTRCD throughout the cancer treatment process using newly developed anticancer treatments and imaging tests.

### Definition of CTRCD

Various terminologies and definitions have been used to define CTRCD across guidelines and clinical trials [22]. This discrepancy in definitions has resulted in diagnostic and treatment disparities [11]. In the expert consensus paper from the imaging societies including the European Association of Cardiovascular Imaging/American Society of Echocardiography (EACVI/ASE), CTRCD was defined as a decrease in the left ventricular ejection fraction (LVEF) > 10 percentage points to a value < 53% (normal reference value for two-dimensional [2D] TTE) in 2014 [23]. However, in the 2016 ESC Cardio-Oncology Position Paper, CTRCD was defined as any decrease in LVEF to < 50% or a > 10% reduction from baseline to less than the lower limit of normal [24]. In recent oncology society guidelines, such as the European Society of Medical Oncology (ESMO) consensus, CTRCD was defined as an absolute LVEF decrease > 10% to < 50% or an absolute LVEF > 20% or symptomatic heart failure (HF). In such cases, cardioprotective therapy and first-line chemotherapy with cardio-oncology input and/or noncardiotoxic second-line cancer treatments should be considered [25]. Considering that LVEF has a low sensitivity to subclinical changes in heart function brought on by early myocyte damage caused by cardiotoxic treatments, LV global longitudinal strain (GLS) has been introduced to detect CTRCD. In the ESMO guideline, normal LVEF with a decrease in average GLS from baseline assessment (≥ 12% relative decrease or ≥ 5% absolute decrease) was recommended as a threshold for initiation of cardioprotective treatments. Meanwhile, the ESC and EACVI/ASE position statements defined CTRCD as a relative reduction in GLS > 15% from baseline.

To clarify the definition of CTRCD, the ESC 2022 Guidelines standardized the definition of CTRCD and introduced a classification system dividing it into symptomatic and asymptomatic categories [10]. Symptomatic CTRCD is further categorized into mild, moderate, severe, and very severe according to the need for HF treatment. Asymptomatic CTRCD is classified as mild, moderate, or severe, with mild CTRCD defined as LVEF ≥ 50% and a new relative decrease in GLS by > 15% from baseline and/or a new increase in levels of cardiac biomarkers. Severe CTRCD is defined as a new LVEF reduction to < 40%. Moderate CTRCD is defined as a new LVEF reduction by ≥ 10 percentage points to a value of 40% to 49% or a new LVEF reduction by < 10 percentage points to a value of 40% to 49%, combined with either a new relative decrease in GLS by > 15% from baseline or a new increase in cardiac biomarker levels.

**Figure:**
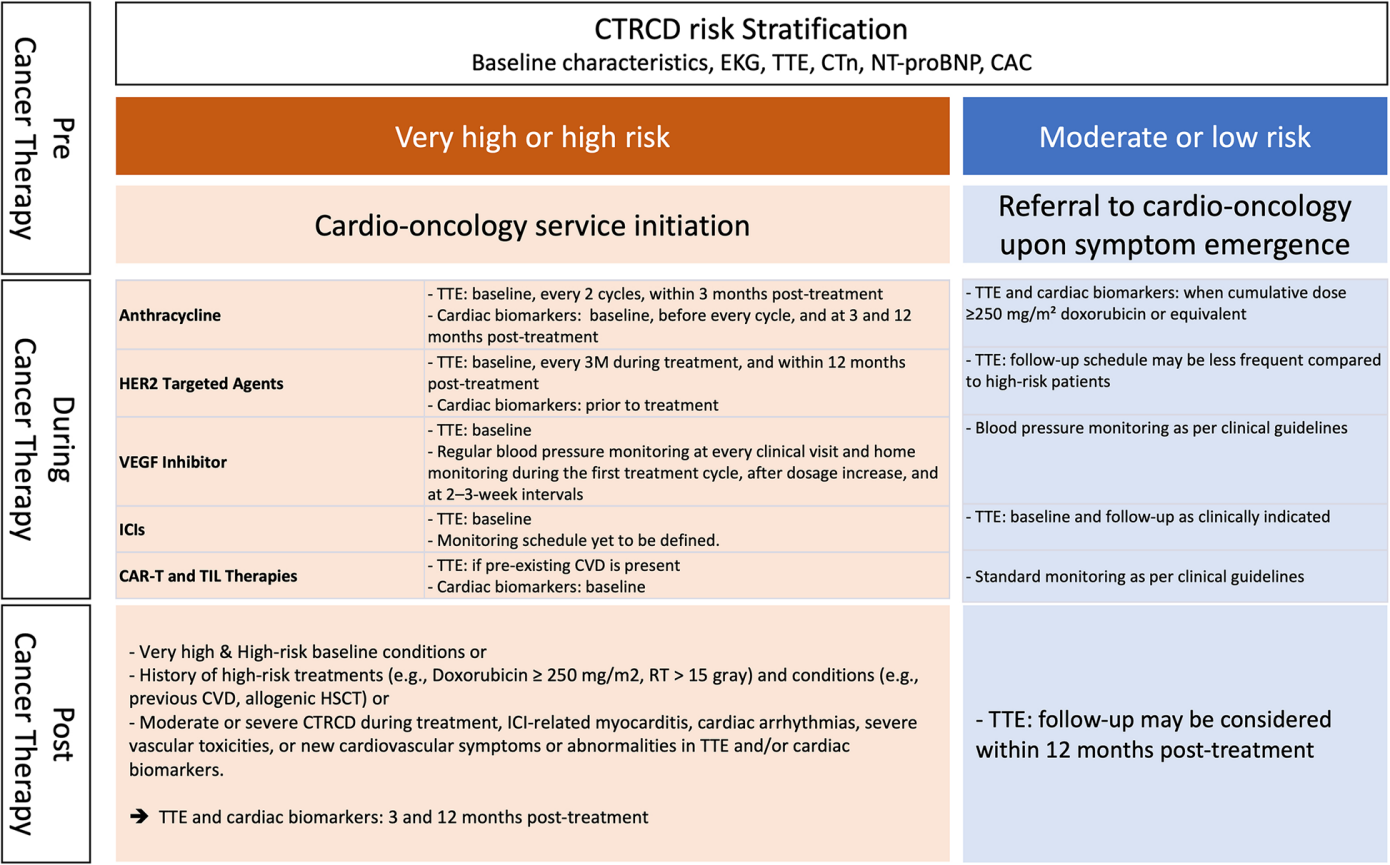
Central illustration Coronary artery calcification information from noncardiac computed tomography acquired from cancer staging workups could be used for cancer therapy-related cardiac dysfunction (CTRCD) risk stratification. EKG, electrocardiography TTE, transthoracic echocardiography; cTn, cardiac troponin; NT-proBNP, N-terminal pro-brain natriuretic peptide; CAC, coronary artery calcium score; VEGF, vascular endothelial growth factor; ICI, immune checkpoint inhibitor; CAR-T, chimeric antigen receptor T-cell; TIL, tumor-infiltrating lymphocyte; RT, radiation therapy; CVD, cardiovascular disease; HSCT, hematopoietic stem cell transplantation

Taken together and considering the measurement variability in 2D TTE (up to 10%) and agreement across professional guidelines, these results underlie a definition of CTRCD as a decrease in LVEF greater than 10% from baseline (Table 1). In cases where the follow-up LVEF is ≥ 50%, a reduction in GLS of > 15% from baseline or a new increase in cardiac biomarker levels should be present to avoid unnecessary alterations or cessation of cancer treatment. In addition, a relative decrease in LV GLS > 15% from baseline should be defined as CTRCD regardless of the change in LVEF. In addition, since an LVEF less than 40% is HF with reduced ejection fraction and requires HF treatment, any new LVEF reduction below 40% should be considered as CTRCD. Finally, patients exhibiting any symptoms or signs that meet HF diagnosis criteria according to the 2021 ESC Guidelines for the diagnosis and treatment of acute and chronic HF should also be classified as CTRCD [26]. Cardinal symptoms for HF include shortness of breath, ankle swelling, and fatigue, while signs of HF include elevated jugular venous pressure, pulmonary crackles, and peripheral edema. In addition, for individuals who exhibit a decrease in LVEF > 10% and relative reduction in GLS < 15% from baseline or with LVEF ≥ 50%, short-term TTE should be performed within 3 months. This approach is recommended as these patients are considered at elevated risk of CTRCD.

**Table 1:** Definition of chemotherapy-related cardiac dysfunction

No	Definition of CTRCD
1	LVEF reduction by ≥ 10 percentage points from baseline (if LVEF ≥ 50%, GLS decline by > 15% from baseline and/or new rise in cardiac biomarkers^a,b^)
2	Relative decline in GLS by > 15% from baseline regardless of LVEF change
3	New LVEF reduction to < 40%
4	Any symptom and sign of heart failure with LVEF and supportive diagnostic biomarkers^c^

*CTRCD* Cancer therapy-related cardiac dysfunction, *LVEF* Left ventricular ejection fraction, *GLS* Global longitudinal strain

^a^Cardiac troponins: 99th percentile, B-type natriuretic peptide ≥ 35 pg/mL, N-terminal pro-brain natriuretic peptide ≥ 125 pg/mL or new significant rise from baseline beyond the biological and analytical variation of the assay used

^b^For individuals who exhibit a decrease in LVEF > 10% and relative reduction in GLS < 15% from the baseline, and LVEF ≥ 50%, it is advisable to conduct a short-term transthoracic echocardiography within 3 months. This approach is recommended as these patients are considered at elevated risk for progressing to cancer therapy-related cardiac dysfunction

^c^Cardinal symptoms for heart failure include shortness of breath, ankle swelling, and fatigue, while signs of heart failure include elevated jugular venous pressure, pulmonary crackles, and peripheral edema

### Risk stratification for CTRCD before anticancer treatment

The risk of CTRCD varies depending on a patient's clinical characteristics and cancer treatment [7, 10]. Therefore, CTRCD prevention should be individualized and initiated at the time of cancer diagnosis, even before the start of cancer therapy; this is a class I indication in the 2022 ESC Guidelines [10]. This allows cardio-oncology services to plan anticancer treatment, a CTRCD surveillance schedule, and a CTRCD prevention strategy based on CTRCD risk of patients.

A specified CTRCD risk stratification system is required for individualized CTRCD risk stratification of cancer patients. However, a limited number of risk stratification systems is available for CTRCD risk assessment. Furthermore, most of these scores were developed for specific subsets of cancer patients (e.g., breast cancer), making it difficult to generalize or extrapolate their results to other forms of malignancy [27, 28]. Among the available risk stratification systems, the 2022 ESC Guidelines recommends using the Heart Failure Association–International Cardio-Oncology Society (HFA-ICOS) risk assessment algorithm. This system includes cardiovascular toxicity based on patient baseline characteristics, such as age, hypertension, chronic kidney disease, proteinuria, diabetes, dyslipidemia, history of previous cardiovascular disease (CVD), electrocardiography, TTE assessment (LVEF, LV hypertrophy), and cardiac serum biomarkers including cardiac troponin (cTn) and N-terminal pro-brain natriuretic peptide (NT-proBNP). Table 2 summarizes the very high- and high-risk findings based on the HFA-ICOS algorithm. Recognizing patients with very high- or high-risk conditions for CTRCD is crucial. For these individuals, it is recommended to introduce cardio-oncology services even before starting cancer treatment. It is essential to weigh the advantages and disadvantages of cardiotoxic anticancer treatments for these patients and to consider cardioprotective measures. In contrast, for those with moderate or low CTRCD risk, a referral to cardio-oncology can be made at CTRCD onset. For example, the factors that typically indicate a moderate risk of cardiovascular toxicity during most cancer therapies include an LVEF between 50 to 54%, elevated baseline level of cTn or NT-proBNP, age of 65 to 79 years, hypertension, chronic kidney disease, diabetes mellitus, current or significant history of smoking, prior exposure to radiation therapy, or obesity with a body mass index exceeding 30 kg/m^2^.

**Table 2:** Summary of very high- and high-risk finding for cardiotoxicity during cancer therapy based on the HFA-ICOS risk assessment algorithm

Risk of cardiotoxicity	Finding
Very high risk	Previous history of heart failure, cardiomyopathy, or CTRCD HER2-targeted therapies: previous exposure to trastuzumab VEGF inhibitors: MI or PCI or CABG or stable angina VEGF inhibitors, BCR-ABL inhibitors, multiple myeloma therapies: arterial vascular disease Multiple myeloma therapies: venous thrombosis, prior proteasome inhibitors cardiovascular toxicity, cardiac amyloidosis
High risk	LVEF < 50% Age ≥ 80 yr VEGF inhibitors, BCR-ABL inhibitors, and multiple myeloma therapies: age ≥ 75 yr Anthracycline chemotherapy, HER2-targeted therapies, and RAF/MEK inhibitors: severe valvular heart disease or MI or PCI or CABG or stable angina Anthracycline therapy, VEGF inhibitors, multiple myelomas, and RAF/MEK inhibitors: previous exposure to anthracycline, RT to left chest or mediastinum BCR/ABL inhibitors: abnormal ankle-brachial index, pulmonary hypertension, CVD 10-yr risk score > 20%, current smoker, or significant smoking history VEGF inhibitors: venous thrombosis VEGF inhibitors or BCR-ABL inhibitors: QTc ≥ 480 ms Multiple myeloma therapies: prior immunomodulatory drugs cardiovascular toxicity, elevated baseline NP for

*HFA-ICOS* Heart Failure Association–International Cardio-Oncology Society, *CTRCD* Cancer therapy-related cardiac dysfunction, *VEGF* Vascular endothelial growth factor, *MI* Myocardial infarction, *PCI* Percutaneous coronary intervention, *CABG* Coronary artery bypass grafting, *BCR-ABL* Breakpoint cluster region-Abelson oncogene, *LVEF* Left ventricular ejection fraction, *RAF* Rapidly accelerated fibrosarcoma, *MEK* Mitogen-activated extracellular signal-regulated kinase, *RT* Radiation therapy, *CVD* Cardiovascular disease, *QTc* Corrected QT interval, *NP* Natriuretic peptide

### Transthoracic echocardiography

As recommended in the current professional guidelines, additional information from cardiovascular imaging contributes to CTRCD risk stratification at baseline. TTE is the preferred imaging modality for baseline risk stratification because it provides quantitative assessment of LV systolic and diastolic function, significant valve disease, and coronary artery disease by abnormal wall motion. Furthermore, given that decreasing LVEF and LV GLS are the core of CTRCD diagnosis, baseline TTE information serves as a reference for subsequent follow-up studies.

### Coronary artery calcium score

Recently, coronary artery calcium score (CACS) acquired by electron beam CT or multidetector CT has been widely used in the asymptomatic general population for cardiovascular risk stratification [29]. There has been an attempt to incorporate CACS into risk stratification for CTRCD in cancer patients and long-term CVD risk assessment in cancer survivors. Given that most cancer patients underwent noncardiac chest CT or radiation therapy (RT) planning CT for staging and treatment planning, CACS by noncardiac CT may be useful for risk stratification and assessment of CV. Recent studies have shown that increased CACS on chest CT or RT planning CT is associated with future CVD risk [30, 31]. Furthermore, it is feasible to fully automate the evaluation of CACS using sophisticated automated algorithms or machine-learning technology [30–34]. However, given that the evidence of future CTRCD and CVD risk assessment with CACS using noncardiac CT has been primarily developed and validated in breast cancer patients with a focus on long-term CVD risk prediction and not CTRCD during cancer therapy, additional evidence for the use of CACS for prediction of CTRCD during anticancer treatment is required.

### Coronary CT angiography

In addition, given the ability to visualize coronary luminal stenosis and plaque characteristics, coronary CT angiography (CCTA) has been widely used in the diagnosis of coronary artery disease and might have a role in the risk stratification of CTRCD in cancer patients [35, 36]. However, studies have not demonstrated any additional prognostic value of CCTA for predicting future cardiovascular events in asymptomatic individuals [37, 38]. Furthermore, the use of contrast agents and higher doses of radiation in CCTA compared to CACS. Because of this, asymptomatic cancer patients should not undergo CCTA for risk stratification purposes. Table 3 summarizes the diagnostic modalities recommended as class I indications in the 2022 ESC Guidelines.

**Table 3:** Summary of recommended baseline screening tests for chemotherapy

Chemotherapy category	Necessary baseline screening test
Anthracycline, HER2-targeted therapy	TTE (all patients) NT-proBNP (for patients at very high risk) cTn (for patients at very high risk)
Fluoropyrimidine	TTE (for patients with previous CVD)
VEGF, RAF/MEK, and BTK inhibitors	TTE (for patients at very high risk)
ICI	TTE (for patients at very high risk) NT-proBNP (all patients) cTn (all patients)
CART-T and TIL therapies	TTE (for patients with previous CVD) NT-proBNP (all patients) cTn (all patients)
HSCT	TTE (all patients)
Proteasome inhibitor	TTE (all patients) NT-proBNP (for patients at very high risk)

*TTE* Transthoracic echocardiography, *NT-proBNP* N-terminal pro-brain natriuretic peptide, *cTn* Cardiac troponin, *CVD* Cardiovascular disease, *VEGF* Vascular endothelial growth factor, *RAF* Rapidly accelerated fibrosarcoma, *MEK* Mitogen-activated extracellular signal-regulated kinase, *BTK* Bruton tyrosine kinase, *ICI* Immune checkpoint inhibitor, *CAR-T* Chimeric antigen receptor T-cell, *TIL* Tumor-infiltrating lymphocyte, *HSCT* Hematopoietic stem cell transplant

### Monitoring of CTRCD during anticancer therapy

The optimal monitoring strategy for CTRCD, including diagnostic modality and schedule, can vary depending on various factors, such as the type of chemotherapy drug and dose and patient risk for CTRCD. The monitoring protocol for patients undergoing potential cardiotoxic anticancer therapy is tailored to their baseline risk for CTRCD and should involve a combination of clinical evaluation, cardiac biomarkers, and TTE. TTE has been extensively used in the surveillance of LV function based on LVEF and LV GLS. In addition, cTn and NT-proBNP can aid in identifying patients at risk for or experiencing cardiac damage. The integration of these imaging and biomarker assessments with clinical evaluation offers a reasonably high negative predictive value for the detection of both symptomatic and asymptomatic cardiotoxicities (Table 4).

**Table 4:** Chemotherapy related cardiotoxicity monitoring strategy according to anticancer therapy

Anticancer therapy	Monitoring strategy
Anthracycline	TTE at baseline and within 12 mo post-treatment (for all patients) TTE every two cycles and within 3 months post-treatment (for high- and very high-risk patients) cTn and NT-proBNP at baseline, before every cycle, and 3 and 12 mo post-treatment (high- and very high-risk patients) TTE and serum cardiac biomarker follow-up during treatment (for moderate- and low-risk patients) when a cumulative dose of ≥ 250 mg/m^2^ of doxorubicin or equivalent
HER2-targeted agent	TTE at baseline, every 3 mo during treatment, and within 12 mo post-treatment (all breast cancer patients) TTE every 3 mo during the first year and every 6 mo for future treatments (for palliative role) cTn and NT-proBNP (only in high- and very high-risk patients prior to treatment)
VEGF inhibitor	TTE at baseline (only for high- and very high-risk patients) Regular blood pressure monitoring at every clinical visit, home monitoring during the first treatment cycle, after each dosage increase of VEGF inhibitors, and at 2–3-wk intervals
ICI	TTE at baseline (high-risk patients) Exact monitoring schedule: not defined
CAR-T and TIL therapies	Baseline ECG, NT-proBNP, and cTn (for all patients) Baseline TTE (for patients with preexisting CVD)

*TTE* Transthoracic echocardiography, *cTn* Cardiac troponin, *NT-proBNP* N-terminal pro-brain natriuretic peptide, *VEGF* Vascular endothelial growth factor, *ICI* Immune checkpoint inhibitors, *CAR-T* Chimeric antigen receptor T-cell, *TIL* Tumor-infiltrating lymphocyte, *ECG* electrocardiogram, *CVD* Cardiovascular disease

### Anthracycline

Anthracycline is a well-known drug for CTRCD, and anthracycline-induced CTRCD is characterized by a dose-dependent and cumulative process that can present either with or without symptoms. The 2022 ESC Guidelines recommended TTE at baseline and within 12 months after completion of treatment in all patients with anthracycline therapy as a class I indication [10]. In patients with high or very high risk of CTRCD, TTE is recommended every two cycles and within 3 months after completion of chemotherapy. Measurement of cardiac biomarkers including cTn and NT-proBNP is also recommended at baseline, before every cycle during chemotherapy, and 3 and 12 months after completion of chemotherapy as a class I indication. Conversely, in patients with moderate or low risk of CTRCD, TTE and serum cardiac biomarker follow-up during anticancer treatment is recommended as a class II indication only, with a cumulative dose of ≥ 250 mg/m^2^ of doxorubicin or equivalent.

### HER2-targeted agents

In patients with HER2-positive breast cancer, HER2-targeted therapies have been widely used in neoadjuvant, adjuvant, and palliative settings. HER2-targeted therapies have also been used in non–breast cancer patients. Regardless of cancer type, anti-HER2 therapies cause LV dysfunction in 15% to 20% of patients, which can progress to overt HF if not monitored properly. Thus, LV function surveillance including assessment of LVEF and LV GLS is recommended prior to and every 3 months during treatment.

Baseline TTE and follow-up surveillance TTE every 3 months and within 12 months after completing treatment are recommended in most professional guidelines in all breast cancer patients who received HER2-targeted therapy. In patients with palliative HER2-targeted therapy, TTE is recommended every 3 months during the first year, but the surveillance schedule can be reduced to every 6 months for future treatments. Measurement of cardiac biomarkers including cTn and NT-proBNP is recommended only in high- and very high-risk patients prior to anti-HER2-targeted therapies as a class I indication.

### VEGF inhibitor

Angiogenesis inhibitors that target the VEGF signaling pathway are critical in treatments for various types of cancer including renal, thyroid, and hepatocellular carcinomas. However, these novel therapies pose potential cardiotoxic risks, with HF emerging as a particularly concerning complication. Clinical studies indicate that HF occurs in approximately 2% to 4% of patients on bevacizumab (a monoclonal antibody VEGF inhibitor) and 3% to 8% of patients on small-molecule tyrosine kinase inhibitors targeting VEGF receptors [39].

Given that direct cardiotoxicity leading to CTRCD due to VEGF inhibitors is relatively uncommon, baseline TTE is advised as a class I indication exclusively for patients at high or very high risk. Hypertension is one of the most recurrent adverse events tied to VEGF inhibitor treatments. Rapid identification and control of hypertension are crucial to avoid cardiovascular complications, such as CTRCD. The ESC guidelines strongly endorse regular blood pressure monitoring at every clinical visit for patients receiving VEGF inhibitors, bevacizumab, or ramucirumab treatments; this is classified as a class I recommendation. In addition, these guidelines encourage home blood pressure monitoring during the first treatment cycle, after each dosage increase of VEGF inhibitors, and at 2- to 3-week intervals, thereafter, also denoted as a class I recommendation.

### Immune checkpoint inhibitors

ICIs have revolutionized the treatment landscape for various metastatic cancers, offering significant improvements in patient outcomes and survival rates [40]. ICIs are a groundbreaking class of therapies that utilize antibodies to block inhibitory proteins like cytotoxic T lymphocyte-associated protein-4 (CTLA-4), programmed cell death protein-1 (PD-1), and PD-1 ligand (PD-L1), all of which negatively regulate the T-cell immune response. By inhibiting these "checkpoints," ICIs ramp up the immune response, enabling the immune system to more effectively target and destroy cancer cells [41]. However, this enhanced immune activation can also lead to a range of immune-related toxicities, including cardiovascular complications [42].

Despite its relatively rare occurrence, ICI-mediated fulminant myocarditis is associated with a high mortality rate, ranging from 25 to 50% [43]. Without fulminant myocarditis, recent studies have indicated that ICI may also elevate the incidence of CTRCD when used concurrently with anthracycline [44]. Other cardiovascular toxicities linked to ICI therapy include pericardial disease, vasculitis including temporal arteritis, and noninflammatory HF [13]. The exact mechanisms of these complications are not fully understood, but they likely result from an overactive immune response affecting noncancerous heart tissue [41]. High-risk patients, such as those receiving dual ICIs, combination ICI-cardiotoxic therapy, ICI-related noncardiovascular events, or prior CTRCD or CVD, may benefit from baseline TTE screening [10]. However, the optimal timing and duration for this surveillance remain undefined.

### CAR-T and TIL therapies

Chimeric antigen receptor T-cell (CAR-T) therapy has been increasingly recognized as a viable treatment option for conditions like acute lymphocytic leukemia, aggressive B-cell lymphomas, and, potentially, solid tumors [45]. However, it has been linked to a range of cardiovascular toxicities, such as LV dysfunction, HF, cardiac arrhythmias, pericardial effusion, Takotsubo syndrome, and cardiac arrest [10]. These adverse events, often associated with cytokine release syndrome (CRS), can be serious [46]. CRS can present as fever, rapid breathing and heart rate, low blood pressure, low oxygen level, and/or end-organ dysfunction shortly after treatment. Tumor-infiltrating lymphocyte (TIL) therapies are another promising treatment for late-stage metastatic melanoma [47] though they have potential cardiovascular risks, primarily stemming from direct myocardial and vascular toxicity. Measurement of NP, cTn, and TTE is recommended in patients who develop CRS of American Society for Transplantation and Cellular Therapy (ASCT) grade ≥ 2 [48]. For all patients undergoing CAR-T or TIL therapies, baseline electrocardiogram, NP, and cTn are recommended in all patients before starting therapies; baseline TTE only is recommended in patients with preexisting CVD before starting CAR-T and TIL therapies as class I indications in the 2022 ESC Guidelines.

While the guidelines provide specific recommendations for monitoring schedules and tests, it is essential to assess cost-effectiveness and practicality in large prospective trials. Moreover, future studies should investigate ethnic differences to ensure the applicability of these guidelines across diverse populations.

### Follow-up for CTRCD and chronic cardiovascular complications in cancer survivors

Cardiovascular health is an essential part of post-cancer care due to the potential cardiotoxic effects of both chemotherapy and RT [49–52]. The CTRCD and chronic cardiovascular complications in cancer survivors necessitate comprehensive and tailored follow-up plans for screening and monitoring. The 2022 ESC Guidelines classified cardiovascular risk at the end-of-cancer therapies using both HFA-ICOS assessment and the cumulative dose of cardiotoxic agents and RT [10]. High-risk conditions are included in high and very high baseline cardiovascular toxicity risk based on HFA-ICOS assessment (Table 2). The following anticancer treatments also were included as high-risk conditions: doxorubicin ≥ 250 mg/m^2^; RT > 15 Gy mean heart dose; both doxorubicin ≥ 100 mg/m^2^ and RT 5–15 Gy mean heart dose; and high-risk hematopoietic stem cell transplantation (HSCT) patients with allogenic HSCT, preexisting CVD or multiple uncontrolled cardiovascular risk factors, cancer treatment history (mediastinal or mantle field radiation, alkylating agents, > 250 mg/m^2^ doxorubicin or equivalent), conditioning regimens (total body irradiation, alkylating agents), and development of graft versus host disease. Moderate or severe CTRCD during cancer treatment, ICI-related myocarditis, cardiac arrhythmias, severe vascular toxicities (acute coronary syndrome, stroke, peripheral vascular disease), new cardiovascular symptoms, and new asymptomatic abnormalities in TTE and/or cTn or NT-proBNP at the end of therapy assessment were also considered high-risk conditions for future CVD.

For high-risk asymptomatic patients, it is advisable to conduct TTE and cardiac biomarker measurements at 3 and 12 months following completion of cancer treatments [11]. Similar screenings are suggested within 12 months post-treatment completion for both moderate- and low-risk asymptomatic patients, with the degree of risk based on cardiovascular toxicity baseline risk stratification.

### Evidence from the Korean population regarding cardio-oncology

Regrettably, there is a lack of prospectively or systematically collected data on CTRCD in the Korean (Republic of) population. Most existing data are derived from retrospective, single-center studies [30, 32, 44, 53] or rely on the Korean National Health Insurance Service data [54–59] or the Korea National Health and Nutrition Examination Survey IV–VI [60]. Consequently, it is challenging to postulate the specific CTRCD risk during chemotherapy or the risk for cancer survivors within the Korean population. Despite these limitations, several research findings merit attention.

In terms of the epidemiology of preexisting CVD and new-onset CVD during cancer therapy, one study reported that approximately 11% of patients had preexisting CVD at cancer diagnosis, with around 16% developing new-onset CVD, primarily within the first 5 years postdiagnosis [59]. To better identify patients at high risk for CVD during cancer therapy, the clinical utility of deep learning-based, fully automated CACS software has been a vital tool for pinpointing those at high risk, signifying a bright future for tech-assisted healthcare [30]. For CTRCD assessment during cancer therapy, research has indicated a significant escalation in CTRCD risk when ICIs are used alongside conventional cardiotoxic drugs like doxorubicin [44]. More specifically, sarcoma patients receiving doxorubicin and ICIs had a higher incidence of confirmed and probable CTRCD than those treated solely with doxorubicin. To mitigate CTRCD risk during cancer treatment, studies have demonstrated that adherence to antihypertensive medication is crucially linked to reduced overall and cardiovascular mortality, emphasizing the need for comprehensive healthcare for such patients [54].

For long-term cancer survivors, especially lung cancer survivors, Korean studies have shown increased risk of comorbid CVD, which can be exacerbated by factors such as hypertension and sedentary lifestyle [53]. The importance of modifiable CVD risk factors has been highlighted, with cancer survivors showing a higher 10-year probability of CVD, particularly those with hepatic, colon, lung, breast, and gastric cancers [60]. In addition, the triglyceride-glucose index “loge (fasting triglyceride [mg] × fasting glucose [mg] / 2)” is a straightforward surrogate marker for risk of future CVD events, particularly atherosclerotic conditions, in cancer survivors [55]. Recent research also suggests that increased physical activity post-cancer diagnosis is inversely related to CVD risk, and that even modest enhancements in physical activity can offer substantial health benefits for cancer survivors [56].

These insights emphasize the imperative need for a coordinated cardio-oncological approach in both treatment and long-term care of cancer patients in Korea. This approach should prioritize regular cardiovascular monitoring and management for those undergoing cancer therapies and long-term survivors with a history of high-risk cancer. To address this need, the Working Group on Cardio-Oncology of the Korean Society of Cardiology is conducting an extensive big data analysis to assess CTRCD risk during and after cancer therapy. We anticipate that forthcoming publications will provide data more specifically tailored to the Korean population.

## Conclusions

Understanding and addressing CTRCD are vital in optimizing the cardiovascular health of cancer patients and survivors, necessitating systematic follow-up strategies incorporating cardiovascular imaging and cardiac biomarkers for effective prediction, monitoring, and management of cardiovascular toxicities associated with anticancer treatments.

## Data Availability

Not applicable.

## Abbreviations

ASE: American Society of Echocardiography
CACS: Coronary artery calcium score
CAR-T: Chimeric antigen receptor T-cell
CCTA: Coronary computed tomography angiography
CRS: Cytokine release syndrome
CT: Computed tomography
CTLA-4: Cytotoxic T lymphocyte-associated protein-4
cTn: Cardiac troponin
CTRCD: Cancer therapy-related cardiac dysfunction
CVD: Cardiovascular disease
EACVI: European Association of Cardiovascular Imaging
EHA: European Hematology Association
ESC: European Society of Cardiology
ESMO: European Society of Medical Oncology
ESTRO: European Society for Therapeutic Radiology and Oncology
GLS: Global longitudinal strain
HFA-ICOS: Heart Failure Association–International Cardio-Oncology Society
HF: Heart failure
HSCT: Hematopoietic stem cell transplantation
ICI: Immune checkpoint inhibitor
ICOS: International Cardio-Oncology Society
LV: Left ventricular
LVEF: Left ventricular ejection fraction
NP: Natriuretic peptide
NT-proBNP: N-terminal pro-brain natriuretic peptide
PD-1: Programmed cell death protein-1
PD-L1: Programmed cell death protein-1 ligand
RT: Radiation therapy
TIL: Tumor-infiltrating lymphocyte
TTE: Transthoracic echocardiography
VEGF: Vascular endothelial growth factor
